# *Staphylococcus aureus* Leukocidin A/B (LukAB) Kills Human Monocytes via Host NLRP3 and ASC when Extracellular, but Not Intracellular

**DOI:** 10.1371/journal.ppat.1004970

**Published:** 2015-06-12

**Authors:** Jason H. Melehani, David B. A. James, Ashley L. DuMont, Victor J. Torres, Joseph A. Duncan

**Affiliations:** 1 Department of Pharmacology, University of North Carolina at Chapel Hill, Chapel Hill, North Carolina, United States of America; 2 Department of Microbiology, New York University School of Medicine, New York, New York, United States of America; 3 Department of Medicine, Division of Infectious Diseases, University of North Carolina at Chapel Hill, Chapel Hill, North Carolina, United States of America; 4 Lineberger Comprehensive Cancer Center, University of North Carolina at Chapel Hill, Chapel Hill, North Carolina, United States of America; Johns Hopkins School of Medicine, UNITED STATES

## Abstract

*Staphylococcus aureus* infections are a growing health burden worldwide, and paramount to this bacterium’s pathogenesis is the production of virulence factors, including pore-forming leukotoxins. Leukocidin A/B (LukAB) is a recently discovered toxin that kills primary human phagocytes, though the underlying mechanism of cell death is not understood. We demonstrate here that LukAB is a major contributor to the death of human monocytes. Using a variety of *in vitro* and *ex vivo* intoxication and infection models, we found that LukAB activates Caspase 1, promotes IL-1β secretion and induces necrosis in human monocytes. Using THP1 cells as a model for human monocytes, we found that the inflammasome components NLRP3 and ASC are required for LukAB-mediated IL-1β secretion and necrotic cell death. *S*. *aureus* was shown to kill human monocytes in a LukAB dependent manner under both extracellular and intracellular *ex vivo* infection models. Although LukAB-mediated killing of THP1 monocytes from extracellular *S*. *aureus* requires ASC, NLRP3 and the LukAB-receptor CD11b, LukAB-mediated killing from phagocytosed *S*. *aureus* is independent of ASC or NLRP3, but dependent on CD11b. Altogether, this study provides insight into the nature of LukAB-mediated killing of human monocytes. The discovery that *S*. *aureus* LukAB provokes differential host responses in a manner dependent on the cellular contact site is critical for the development of anti-infective/anti-inflammatory therapies that target the NLRP3 inflammasome.

## Author Summary

*Staphylococcus aureus* infections are becoming increasingly common, aggressive, and difficult to manage clinically. *S*. *aureus* produces a number of pore-forming toxins that target and kill immune cells. In this study, we demonstrate that LukAB is primarily responsible for *S*. *aureus*-mediated targeting and killing of human monocytes. We show that the NLRP3-ASC inflammasome, a sensor of cell membrane damage and trigger of inflammation, is critical for this response. *S*. *aureus* uses LukAB to kill immune cells both through external interactions (LukAB on the cell surface) and through internal interactions (LukAB secretion after *S*. *aureus* is engulfed by the immune cell). Interestingly, we show that the mechanism by which LukAB kills immune cells in these two settings differs. This is the first report of a *S*. *aureus* toxin manipulating unique immune signaling pathways depending on the cellular site of contact. Understanding the multitude of ways by which *S*. *aureus* evades the immune response is critical for our ability to treat infections with this pathogen.

## Introduction

*S*. *aureus* is one of the most commonly identified causes of infection, and is responsible for a significant health and economic burden including approximately 100,000 life-threatening infections per year in the United States [1]. *S*. *aureus* can cause a variety of diseases that range from recurrent epidermal abscesses to life-threatening necrotizing pneumonias. To promote these infections, *S*. *aureus* produces many different virulence factors including several cytotoxic beta-barrel pore-forming toxins such as: α-toxin (Hla), Leukocidin AB (LukAB), Leukocidin ED (LukED), Panton-Valentine leukocidin (PVL), and gamma hemolysins (HlgAB and HlgCB) [2,3]. Among these toxins, Hla and PVL are the most studied *in vivo*. In mouse studies, Hla has been implicated in enhancing virulence in numerous infectious models, including keratitis, mastitis, pneumonia, skin abscess, and lethal intraperitoneal infection [4–9]. In contrast, variable contributions of PVL to virulence have been demonstrated in mouse models of keratitis, pneumonia, bone and muscle infections [5,10–13]. Rabbit models of infection, however, highlight a clearer role of PVL in *S*. *aureus* virulence [14–17]. Rabbit neutrophils are significantly more susceptible to PVL than mouse neutrophils [18], but remain relatively resistant to the toxin when compared to human neutrophils, which is due to the species selectivity of PVL towards its cellular receptor, C5aR [19].

The most recently identified *S*. *aureus* leukotoxin is LukAB (also known as LukGH) [20,21]. LukAB kills primary human neutrophils, monocytes, macrophages, and dendritic cells [20]. As with PVL, LukAB also exhibits species specificity towards human leukocytes [22,23]. LukAB binds to CD11b, a component of the CD11b/CD18 integrin (also known as αM/β2, CR3, or Mac-1), to target and kill human neutrophils [22]. A glutamic acid at position 323 within the unique C-terminal region of the LukA subunit binds directly to the I-domain of human CD11b to promote cell binding and subsequent pore-mediated cell lysis [24]. Interestingly, sufficient differences exist between the mouse and human CD11b I-domain to render mouse leukocytes resistant to LukAB [22]. Additionally, *S*. *aureus* escape from phagocytic killing by human neutrophils requires LukAB production [20,22,24,25], suggesting this toxin may play a unique and important role in bacterial survival and persistence.

Host innate immune response to combat *S*. *aureus* involves a diverse set of pattern recognition/danger responsive receptors including the intracellular NOD-like Receptor (NLR) protein 3 (NLRP3) [26]. NLRP3, together with proteins ASC and Caspase 1, form a cytoplasmic oligomeric complex known as the NLRP3 inflammasome, which plays a critical role in initiating innate immune responses [27]. *S*. *aureus* and its secreted toxins Hla, HlgACB, and PVL have all been found to activate the NLRP3 inflammasome in monocytes/macrophages leading to activation of Caspase 1, secretion of Caspase 1-processed pro-inflammatory cytokines IL-1β and IL-18, and induction of necrotic cell death [26,28–31]. In a mouse skin infection model, neutrophil NLRP3 inflammasome activation and IL-1β secretion promotes inflammation and abscess formation that accompany bacterial clearance [32]. This is in contrast to murine *S*. *aureus* pneumonia where the NLRP3-driven response is not required for bacterial clearance but instead exacerbates the severity of disease pathology [26,33].

Herein, we sought to investigate how *S*. *aureus* directly kills human monocytes and whether the NLRP3 inflammasome contributes to this process. We utilized THP1 human monocytic cells as a model to determine the molecular mechanism of cell death. This cell line expresses high levels of NLRP3, ASC, and pro-Caspase 1 and is also extensively used to study inflammasome activation [34,35]. We show that *S*. *aureus* employs LukAB as the predominant toxin during *ex vivo* infection of monocytes to promote necrotic cell death. Purified LukAB is sufficient to activate Caspase 1, induce secretion of IL-1β and IL-18, and cause necrotic cell death. Importantly, these findings were replicated in primary CD14+ human monocytes. Using shRNA knockdowns in THP1 monocytes, we confirmed that these responses were dependent on LukAB binding to its cellular receptor CD11b, which leads to activation of the NLRP3 inflammasome. In contrast to LukAB binding to the host plasma membrane, LukAB secreted from within the phagosome of THP1 cells requires CD11b, but not ASC or NLRP3, to trigger cell death. Together these results provide a greater understanding of an important, yet previously underestimated, *S*. *aureus* virulence factor and the means by which this toxin targets and kills host monocytes.

## Results

### *S*. *aureus* kills human monocytes in a LukAB dependent manner

**Fig 1:**
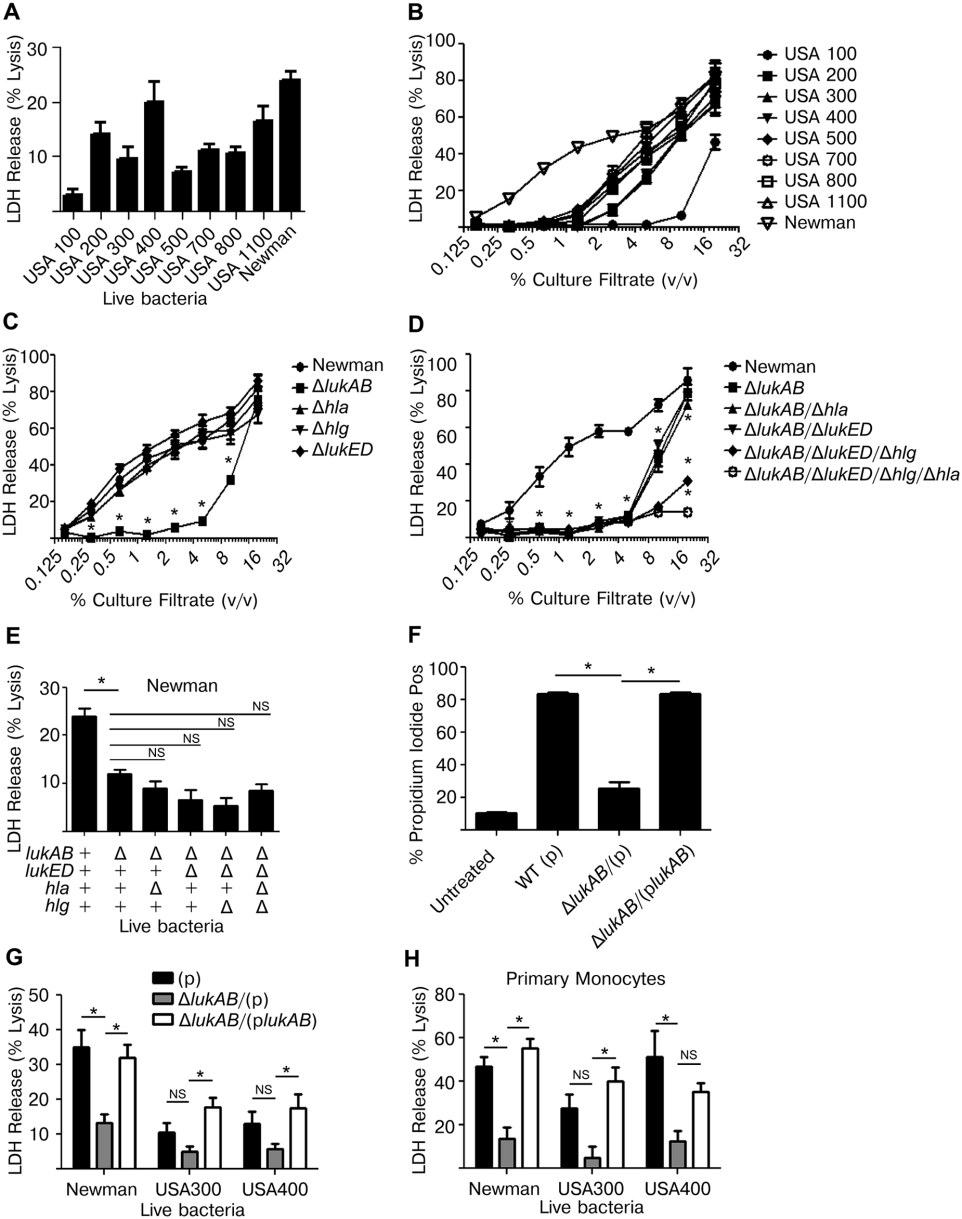
*Staphylococcus aureus* LukAB induces cell death in human monocytic cells. (A) THP1 cells were infected with the indicated *S*. *aureus* strains at a multiplicity of infection (MOI) of 50 for 2 hours and culture supernatants were analyzed for LDH release as a measurement of cell lysis. (B) Culture filtrates, acquired from log-phase growth of *S*. *aureus* strains listed, were used to intoxicate THP1 cells at the indicated concentrations for 4 hours and LDH release was analyzed. (C and D) THP1 cells were intoxicated with culture filtrates from *S*. *aureus* Newman or the indicated isogenic mutants for 4 hours and LDH release was analyzed. (E) THP1 cells were infected with *S*. *aureus* Newman or the indicated isogenic mutants at an MOI of 50 and LDH release was analyzed after 2 hours. (F) THP1 cells were incubated with propidium iodide and intoxicated with culture filtrates from *S*. *aureus* Newman, *lukAB* mutant, or complemented strain at 1% (v/v) for 4 hours then analyzed by flow cytometry. THP1 cells (G) and primary CD14+ human monocytes (H) were infected with *S*. *aureus* Newman, USA300 (LAC) or USA400 (MW2) or the respective *lukAB*-deficient mutants and complemented strains at an MOI of 50 (G) or 25 (H) and LDH release was analyzed after 2 hours. Error bars represent the mean ± standard error of the mean for at least two independent experiments, each performed in triplicate. Experiments with primary cells include at least three independent donors. Asterisks indicate significance at a *p*-value of ≤ 0.05 by Tukey’s multiple comparisons post-test for 1-way or 2-way ANOVA, as appropriate.

To investigate the ability of *S*. *aureus* to kill human monocytes, a variety of live *S*. *aureus* clinical isolates, representing different clonal lineages, were co-cultured with THP1 cells. Each *S*. *aureus* strain killed THP1 cells, albeit to varying degrees, as assessed by the amount of cytoplasmic lactate dehydrogenase (LDH) released into the culture supernatant (Fig 1A). To evaluate the contribution of *S*. *aureus* secreted proteins to cell death, culture filtrates were collected from log-phase grown *S*. *aureus* and used to intoxicate THP1 cells. These culture filtrates were all capable of inducing cell death in a dose dependent manner (Fig 1B).

To further dissect the role of specific secreted toxins, we selected *S*. *aureus* Newman, a well-characterized methicillin-sensitive clinical isolate [36] that exhibited potent cellular killing [20]. Here, THP1 cells were intoxicated with a titration of culture filtrates from *S*. *aureus* Newman or individual isogenic mutants deficient of *lukAB*, *hla*, *hlgACB*, or *lukED* and LDH release was evaluated (Fig 1C). Only loss of LukAB had a significant effect on culture filtrate cytotoxicity, particularly at concentrations of ≤ 5% v/v. Culture filtrates lacking LukAB had residual cytotoxicity at higher concentrations, suggesting that additional factors can also contribute to THP1 cell death (Fig 1C). Using *S*. *aureus* strains lacking multiple toxins and in various combinations, we demonstrated that HlgACB and Hla in culture filtrates also contribute to THP1 killing at these higher concentrations (Fig 1D), consistent with previous reports describing the cytotoxic effects of these toxins on murine cells [30,31,37]. We also utilized these strains to determine the relative effects of each toxin in live *S*. *aureus*-mediated killing of THP1 cells (Fig 1E). Similar to our observations with culture filtrates, loss of LukAB significantly reduced *S*. *aureus* killing of THP1 cells, while the loss of the other toxins had little if any additional effect. To further validate the LDH data, we used propidium iodide (PI), a membrane-impermeable DNA-intercalating dye, to monitor membrane integrity of human monocytes using flow cytometry. In agreement with the LDH release data, LukAB was responsible for the propidium iodide staining observed with culture filtrates of *S*. *aureus* strain Newman (Fig 1F).

*S*. *aureus* Newman naturally does not encode *pvl*, thus we next sought to determine whether LukAB also contributes to THP1 cell death in methicillin-resistant *S*. *aureus* (MRSA) strains that produce PVL. We assessed the cytotoxicity of wildtype and LukAB-deficient strains LAC (USA 300) and MW2 (USA 400), two representative MRSA clones, towards THP1 cells in models of live bacterial infection (Fig 1G). As with Newman, strains LAC and MW2 lacking *lukAB* exhibited reduced cytotoxicity to THP1 cells when compared to the isogenic parental strain, a phenotype complemented by expressing *lukAB* from a plasmid episomally (Fig 1G).

The impact of LukAB in killing human monocytes was also tested using live bacterial infection of purified CD14+ primary human monocytes. The diminished cytotoxicity of *lukAB* deficient *S*. *aureus* strains seen in THP1 experiments was phenocopied in experiments with primary monocytes, as was the complementation of the phenotype by *lukAB*-expressing plasmids (Fig 1H). Thus, these data demonstrate that LukAB is the predominant toxin secreted by *S*. *aureus* to kill human monocytes.

### LukAB potently kills human monocytes by engaging its cellular receptor CD11b

**Fig 2:**
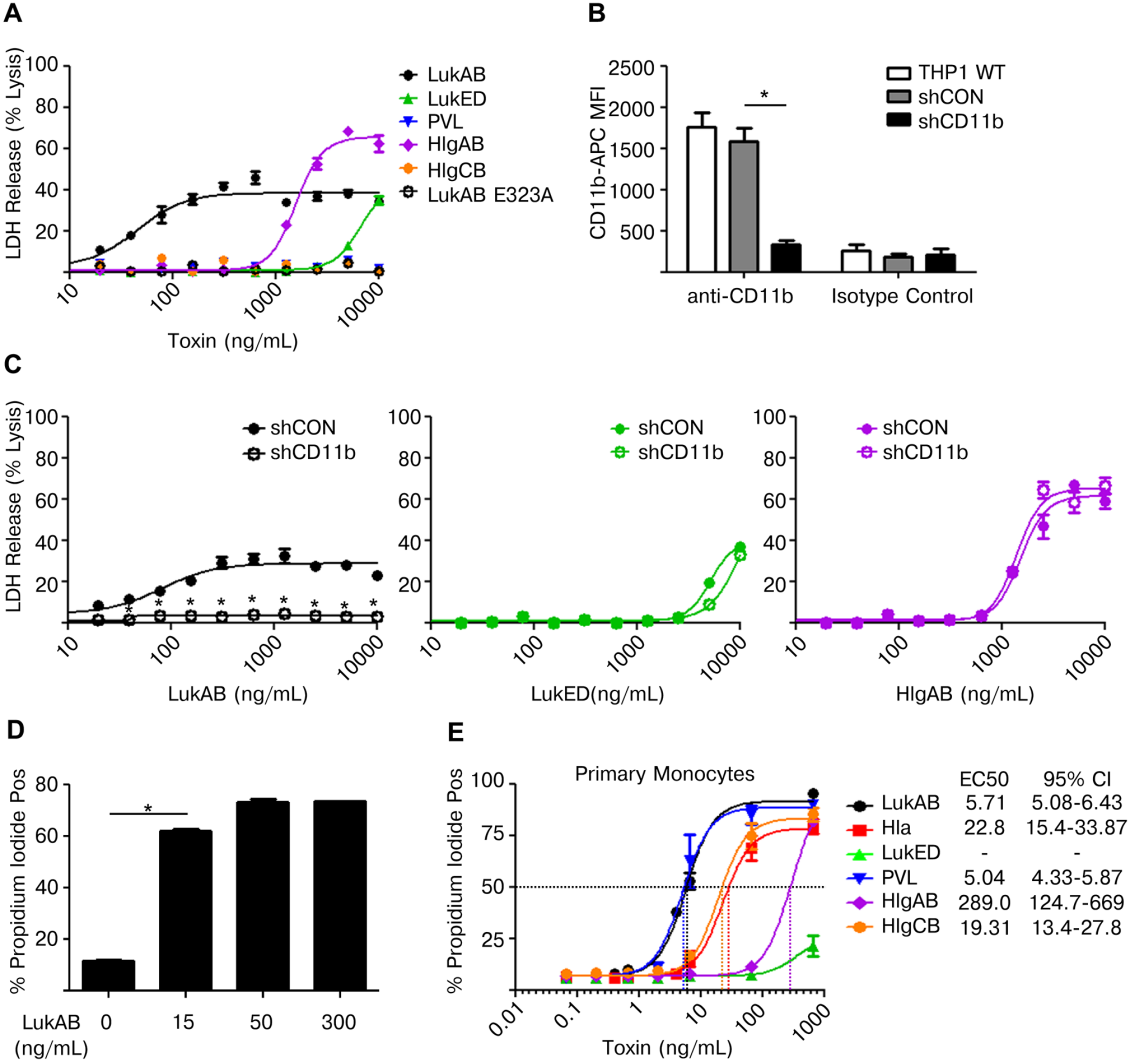
LukAB targets CD11b on human monocytes to potently induce cell death. (A) THP1 cells were intoxicated with titrations of the indicated purified toxins for 1 hour and LDH release was analyzed. (B) THP1 cells were transduced with either non-targeting shRNA or shRNA against CD11b and surface CD11b levels were evaluated by flow cytometry. (C) THP1 cells described in panel B were intoxicated with titrations of the indicated toxins for 1 hour and LDH release was analyzed. (D) THP1 cells were intoxicated with the indicated concentration of LukAB for 1 hour and analyzed by flow cytometry for permeability to propidium iodide. (E) Primary CD14+ human monocytes were intoxicated with titrations of the indicated toxins for 1 hour and analyzed by flow cytometry for permeability to propidium iodide. EC50 values are also shown. Error bars represent the mean ± standard error of the mean for at least two independent experiments, each performed in triplicate. Primary cell experiments include three independent donors. Asterisks indicate significance at a *p*-value of ≤ 0.05 by Tukey’s multiple comparisons post-test for 1-way or 2-way ANOVA, as appropriate.

To determine whether LukAB was sufficient to induce THP1 cell death, and to compare its relative cytotoxicity to that of the other bi-component pore forming toxins, THP1 cells were intoxicated with purified toxins. LukAB, LukED and HlgAB were all able to induce cytotoxicity in THP1 cells, as measured by release of LDH into the culture medium (Fig 2A). In contrast, HlgCB and PVL were unable to lyse THP1 cells [29]. Among the toxins tested, LukAB was the most potent, capable of lysing THP1 cells at concentrations approximately 8- and 12-fold lower than HlgAB and LukED, respectively (Fig 2A).

LukAB targets CD11b on human neutrophils to promote cell death [22], so we next sought to determine if LukAB-CD11b recognition was also required for LukAB activity towards human monocytes. We first utilized a previously characterized LukAB mutant that does not bind CD11b, LukAB E323A [24]. This mutant toxin failed to elicit LDH release from THP1 cells, indicating that CD11b interaction is required for LukAB-mediated death in this monocytic cell line (Fig 2A). To further validate this finding, THP1 cells were transduced to stably express short hairpin RNA (shRNA) against CD11b or a non-targeting control (Fig 2B). Knockdown of CD11b was confirmed through immunostaining and analysis by flow cytometry (Fig 2B). These transduced cells were then intoxicated with purified LukAB, LukED or HlgAB (Fig 2C). The CD11b shRNA knockdown significantly reduced LukAB-mediated cytoxicity, but not cytotoxicity resulting from HlgAB or LukED intoxication, indicating that LukAB recognizes CD11b on human monocytes to induce cell death (Fig 2C). The potency of LukAB in mediating THP1 cell permeability to PI paralleled the potency observed for release of LDH from target cells (Fig 2D).

We next sought to determine the potency to LukAB, relative to other pore-forming toxins in killing CD14+ primary human monocytes. As assessed by PI staining, dose-titrations of each toxin demonstrated that LukAB and PVL were the most potent in their ability to kill primary monocytes (Fig 2E). Interestingly, primary monocytes exhibited a ten-fold increase in susceptibility to LukAB when compared to the THP1 cell line. This difference in toxin susceptibility was accentuated in PVL where primary monocytes were highly susceptible to the toxin while THP1 cells were essentially resistant.

### LukAB-induced cell death displays necrotic features

**Fig 3:**
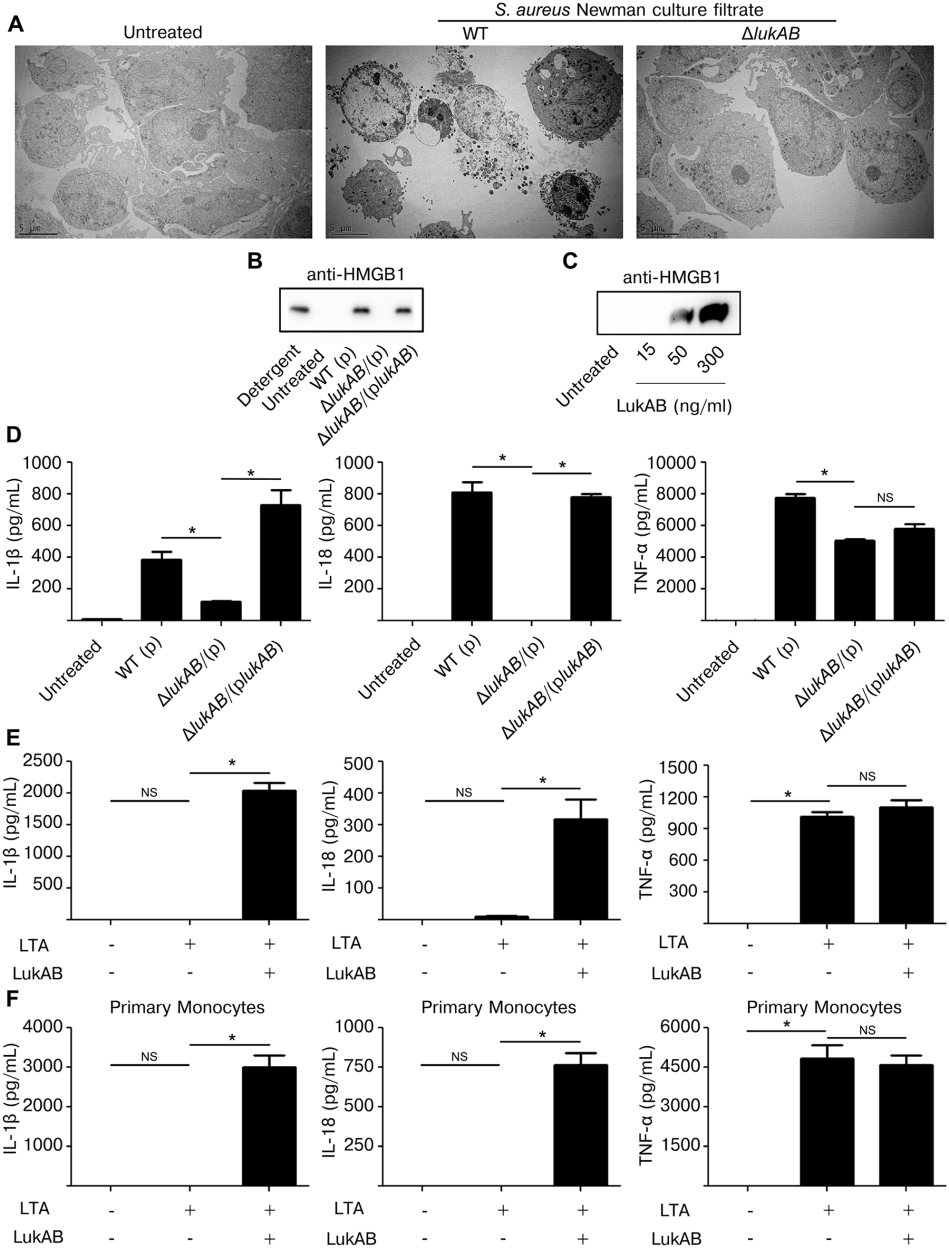
LukAB induces necrotic cell death and secretion of pro-inflammatory cytokines IL-1β and IL-18. (A) THP1 cells were intoxicated with culture filtrate from *S*. *aureus* Newman (10% v/v), isogenic *lukAB* mutant or culture media for 1 hour. Cells were collected, prepared and imaged by transmission electron microscopy (see methods). (B) THP1 cells were intoxicated with 1% (v/v) culture filtrate from *S*. *aureus* Newman or the indicated isogenic mutants and after 4 hours supernatants were collected and analyzed by immunoblot for HMGB1 release. (C) THP1 cells were intoxicated with purified LukAB at the indicated concentrations for 1 hour and supernatants were analyzed by immunoblot for HMGB1 release. (D) THP1 cells were intoxicated with culture filtrates (1% v/v) from the indicated strains and supernatants were collected and analyzed for secretion of the indicated cytokines. THP1 cells (E) and primary CD14+ human monocytes (F) were primed for production of pro-IL-1β with 500 ng/mL LTA for 3 hours followed by intoxication with LukAB (THP1 with 50 ng/mL and CD14+ monocytes with 30 ng/mL) for 1 hour then supernatants were analyzed for secretion of the indicated cytokines. Error bars represent the mean ± standard error of the mean for at least two independent experiments, each performed in triplicate. Primary cell experiments include three independent donors. Asterisks indicate significance at a *p*-value of < = 0.05 by Tukey’s multiple comparisons post-test for 1-way or 2-way ANOVA, as appropriate.

Traditionally, programmed cell death can be morphologically categorized into necrotic or apoptotic phenotypes [38,39]. During necrotic cell death membrane integrity is rapidly lost, releasing cytosolic and nuclear contents into the extracellular milieu. Death with necrotic features leads to inflammation, as cytosolic contents act as endogenous danger signals triggering activation of innate immune signaling. In contrast, apoptosis is thought to be relatively immunologically silent as cytosolic and nuclear contents are broken down into small membrane bound bodies [40]. To further visualize the effect of LukAB on the membrane of THP1 cells, culture filtrates from *S*. *aureus* Newman or the isogenic *lukAB* deficient-mutant were used to intoxicate THP1 cells which were then examined by transmission electron microscopy (Fig 3A). THP1 cells intoxicated with LukAB-containing culture filtrates displayed vacuolation of the cytoplasm, substantial plasma membrane compromise, and gross changes in nuclear morphology, all suggestive of necrotic cell death. This is in contrast to THP1 cells intoxicated with culture filtrates from an isogenic *lukAB* deficient-mutant or with the media control, where membranes were mostly intact and cytoplasmic contents preserved (Fig 3A).

Another marker of necrosis is the release of High Mobility Group Box 1 protein (HMGB1), a nuclear protein that, when released, acts as a pro-inflammatory danger signal [41]. To test whether LukAB induced HMGB1 release, THP1 cells were intoxicated with *S*. *aureus* culture filtrates or purified LukAB and the culture supernatants were evaluated by immunoblot with antibodies specific to HMGB1. We observed that LukAB was necessary and sufficient to induce HMGB1 release (Fig 3B and 3C).

Necrotic cell death paired with secretion of inflammatory cytokines IL-1β and IL-18 is termed pyroptosis [41–43]. To determine if LukAB causes THP1 cells to secrete IL-1β and IL-18, we analyzed culture supernatants from THP1 cells intoxicated with Newman strain culture filtrates (Fig 3D) and purified LukAB (Fig 3E). In this setting, THP1 cells secreted IL-1β, IL-18 and TNF-α in response to Newman strain culture filtrates, though deletion of *lukAB* eliminated secretion of IL-1β and IL-18 with minor effects on TNF-α secretion. THP1 cells do not express pro-IL-1β at baseline and thus require priming with a Toll-like receptor ligand such as lipotechoic acid (LTA) prior to intoxication with purified LukAB. LTA induced TNF-α secretion, but LukAB was required for secretion of IL-1β and IL-18 (Fig 3E). As with THP1 cells, primary CD14+ human monocytes also secreted IL-1β and IL-18 in response to LukAB (Fig 3F).

### LukAB induces activation of Caspase 1 in human monocytes

**Fig 4:**
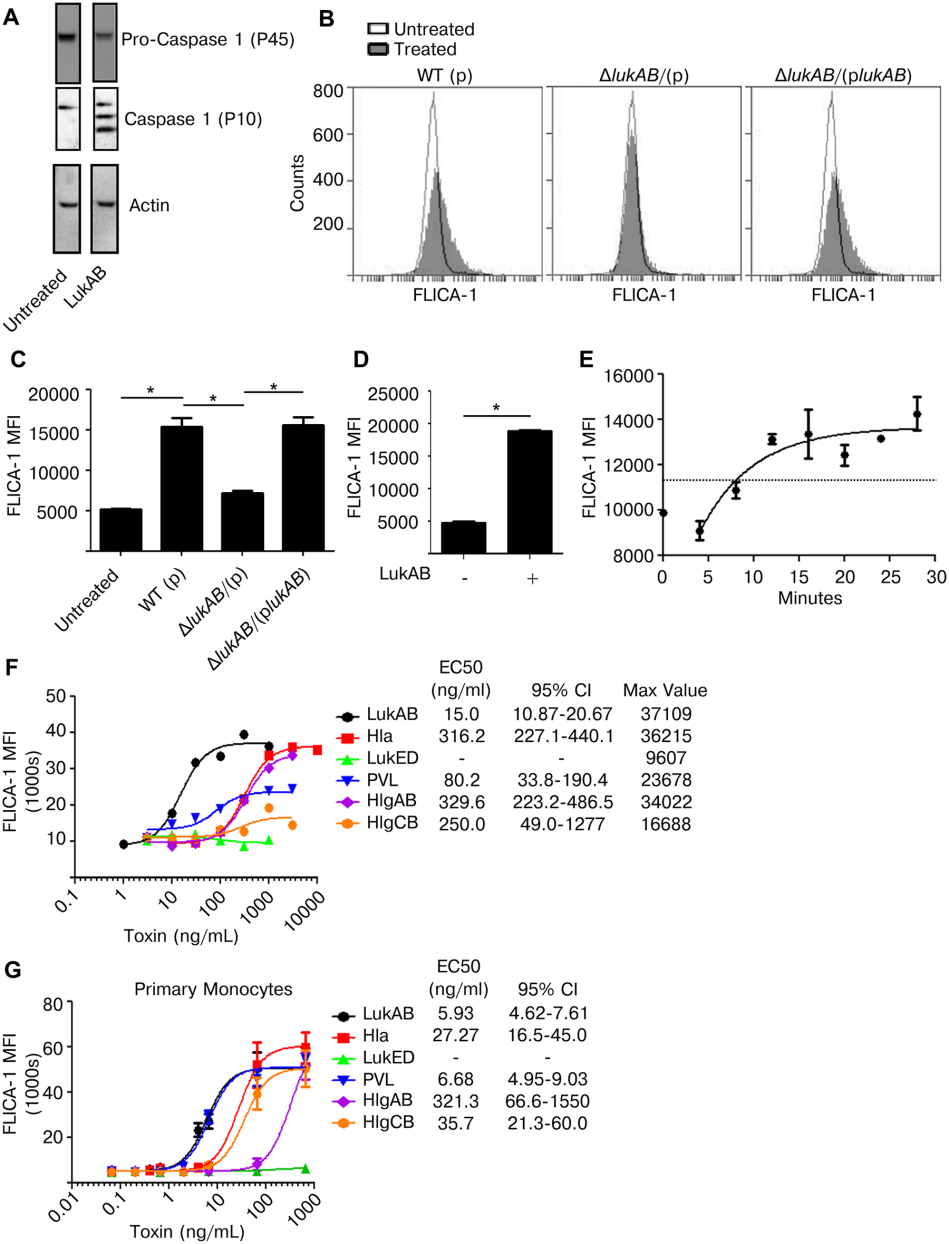
LukAB is a potent activator of Caspase 1. (A) THP1 cells were intoxicated with 50 ng/mL LukAB for 1 hour and cell lysates were analyzed by immunoblot for Caspase 1 cleavage, which indicates activation. (B and C) THP1 cells were incubated with FLICA-1 (660-YVAD-FMK) then intoxicated with culture filtrates (1% v/v) from the indicated *S*. *aureus* stains. After 1 hour, cells were washed, fixed and analyzed by flow cytometry. Panel B shows a representative flow plot from one experiment, and panel C shows the corresponding mean florescence intensities (MFI). (D) THP1 cells were incubated with FLICA-1, intoxicated with purified LukAB (50 ng/mL) for 1 hour, then washed, fixed and analyzed by flow cytometry. (E) FLICA-1 was added to THP1 cells then LukAB (50 ng/mL) was added for time-course samples in descending order. All samples were washed and fixed at the same time, corresponding to different LukAB incubation times, then analyzed by flow cytometry. THP1 cells (F) and primary CD14+ human monocytes (G) were incubated with FLICA-1 and intoxicated with a dose titration of the indicated purified *S*. *aureus* leukotoxins for 1 hour. Cells were washed, fixed and analyzed by flow cytometry. Bars represent the standard error of the mean of triplicate samples. EC50 values are also shown. Each graph is representative of three experiments. Primary cell experiments include three independent donors. Asterisks indicate significance at a *p*-value of ≤ 0.05 by Tukey’s multiple comparisons post-test for 1-way or 2-way ANOVA, as appropriate.

Pyroptosis depends on the activation of Caspase 1 [40]. To determine if LukAB induced Caspase 1 activation, THP1 cells were incubated with purified LukAB and activation of Caspase 1 assessed using immunoblot analyses. The auto-proteolysis-derived P10 subunit, an indication of Caspase 1 activation, was observed in cells treated with purified LukAB (Fig 4A). To quantitatively assess Caspase 1 activation, we used a flow cytometry based fluorescent-labeled peptide inhibitor of Caspase 1 (660-YVAD-FMK) assay (hereafter FLICA-1) [42]. THP1 cells treated with *S*. *aureus* culture filtrates demonstrated a marked increase in FLICA-1 fluorescence when compared to the isogenic *lukAB*–deficient mutant or untreated cells (Fig 4B and 4C). The phenotype of the *lukAB*-deficient mutant could be complemented to that of the WT strain by expressing a plasmid encoding *lukAB*. As observed by immunoblot (Fig 4A), LukAB treatment alone was sufficient to cause enhanced FLICA-1 activation in THP1 cells (Fig 4D). Measurable change in FLICA-1 activation in THP1 cells lagged the introduction of toxin by approximately five minutes (Fig 4E). After the lag period, FLICA-1 activation rapidly increased reaching a plateau over approximately 10 minutes (Fig 4E).

It has previously been reported that Hla and PVL activate Caspase 1 [28,29,37]. We next sought to determine the relative potency of each *S*. *aureus* leukotoxin in inducing Caspase 1 activation as measured by FLICA-1 in both THP1 cells (Fig 4F) and primary CD14+ human monocytes (Fig 4G). In THP1 cells, LukAB was the most potent FLICA-1 activator (Fig 4F). Although PVL did not induce cell lysis in THP1 cells (Fig 2A), it did induce FLICA-1 activation (Fig 4F). In primary human monocytes, LukAB and PVL demonstrated equivalent potency in inducing FLICA-1 activation (Fig 4G).

### ASC and NLRP3 are necessary for LukAB-induced cytokine secretion and necrotic cell death

**Fig 5:**
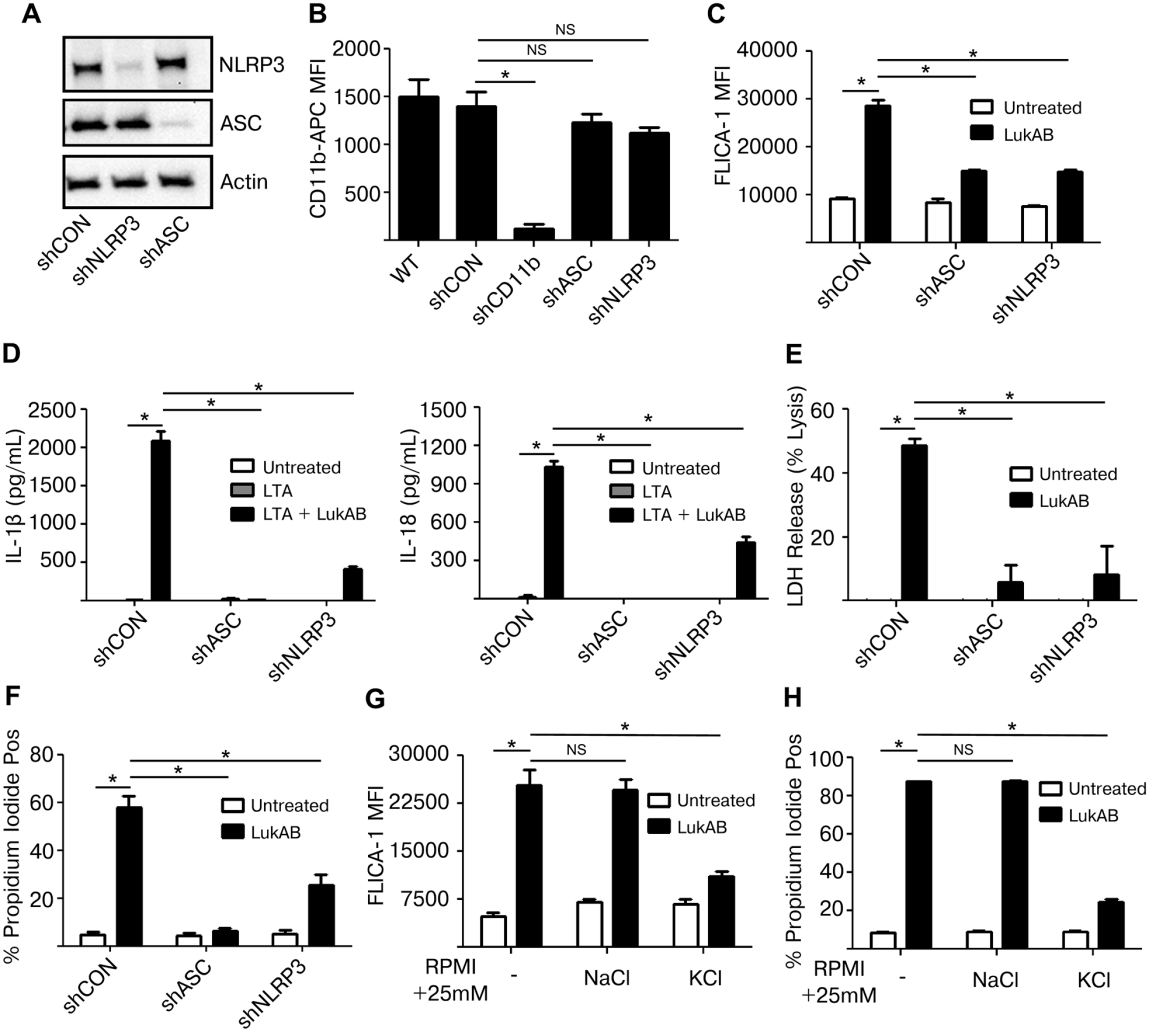
LukAB activates the NLRP3 inflammasome leading to cell death and cytokine secretion. (A) THP1 cells were transduced with shRNA against NLRP3, ASC or a non-targeting control. Cell lysates were analyzed by immunoblot to confirm knock down of NLRP3 and ASC. (B) Surface CD11b levels were evaluated by flow cytometry. (C) THP1 shRNA cells were incubated in the presence of FLICA-1 then intoxicated with LukAB (50 ng/mL) for 1 hour then analyzed by flow cytometry. (D) THP1 shRNA cells were primed with 500 ng/mL LTA for 3 hours followed by intoxication with LukAB (50ng/mL) for one hour. Culture supernatants were collected and analyzed for secretion of the indicated cytokines. (E) THP1 shRNA cells were intoxicated with LukAB (50 ng/mL) for 1 hour and culture supernatants were collected for analysis of LDH release. (F) THP1 shRNA cells were incubated with propidium iodide then intoxicated with LukAB (50 ng/mL) for 1 hour then analyzed by flow cytometry. (G and H) THP1 cells were incubated in media supplemented with an additional 25 mM NaCl or KCl. Cells were incubated with FLICA-1 (G) or propidium iodide (H) then intoxicated with LukAB (50 ng/mL) for 1 hour and analyzed by flow cytometry. Bars represent the mean ± standard error of the mean for at least two independent experiments, each performed in triplicate. Asterisks indicate significance at a *p*-value of ≤ 0.05 by Tukey’s multiple comparisons post-test for 1-way or 2-way ANOVA, as appropriate.

The activation of Caspase 1 by *S*. *aureus* Hla and PVL depends on host NLRP3 and ASC [26,29]. We next sought to determine whether LukAB-mediated Caspase 1 activation, secretion of IL-1β and IL-18 and cell death were also dependent on NLRP3 and ASC. To this end, THP1 cells were transduced to stably express shRNA constructs targeting inflammasome components ASC and NLRP3, or a non-targeting shRNA control. Knock down of ASC and NLRP3 was confirmed by immunoblot analyses (Fig 5A). Importantly, knock down of ASC or NLRP3 did not reduce levels of the LukAB receptor CD11b (Fig 5B). Knock down of ASC or NLRP3 resulted in a significant reduction in LukAB-induced FLICA-1 activation (Fig 5C) and secretion of the Caspase 1 dependent cytokines IL-1β and IL-18 (Fig 5D). Thus, FLICA-1 is a reliable measure of NLRP3 inflammasome activation by LukAB. Moreover, LukAB-induced necrotic cell death as measured by LDH release (Fig 5E) and membrane permeability to PI (Fig 5F) was essentially eliminated when ASC or NLRP3 were depleted. Knock down of ASC or NLRP3 also had a similar effect on IL-1β and IL-18 secretion and cell death in THP1 cells intoxicated with Newman culture filtrates (S1 Fig). Residual cell death and cytokine release was consistently observed in the NLRP3 knock down, a result likely due to incomplete knockdown (Fig 5C–5F).

Activation of the NLRP3 inflammasome involves potassium efflux [43,44], thus we evaluated the role of potassium in LukAB-mediated inflammasome activation. THP1 cultures supplemented with potassium chloride, but not sodium chloride or choline chloride (S2 Fig), inhibited LukAB-induced NLRP3-inflammasome activation as assessed by FLICA-1 activation (Fig 5G) and cell membrane PI permeability (Fig 5H). Altogether, these results indicate that LukAB activates the NLRP3 inflammasome to initiate Caspase 1-dependent cytokine release and necrotic cell death.

### Caspase 1 is required for LukAB-induced cytokine secretion

**Fig 6:**
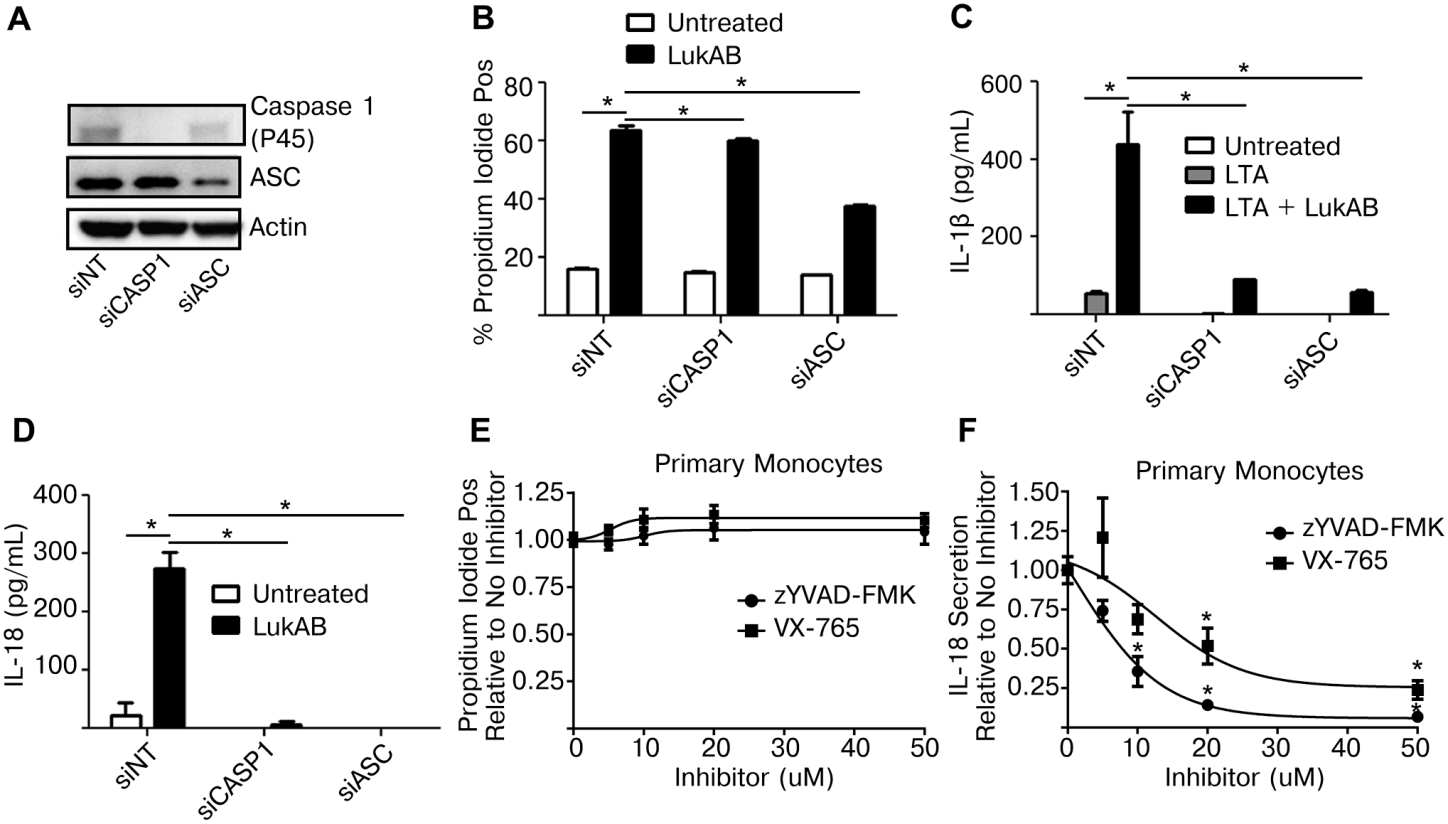
Genetic or pharmacologic disruption of Caspase 1 blocks LukAB-induced cytokine secretion but not cell death. (A) THP1 cells were transfected with siRNA against Caspase 1, ASC or a non-targeting sequence. Cell lysates were collected and analyzed by immunoblot to confirm knockdown of pro-Caspase 1 and ASC. (B) THP1 siRNA cells were incubated with propidium iodide and intoxicated with 50 ng/mL LukAB for 1 hour then analyzed by flow cytometry. (C and D) THP1 siRNA cells were either primed with LTA (500ng/mL) (C) or untreated (D) then intoxicated with LukAB (50ng/mL) for 1 hr before culture supernatants were analyzed for release of IL-1β (C) or IL-18 (D). (E and F) Primary CD14+ human monocytes were incubated with the indicated concentration of z-YVAD-FMK or VX-765 for 30 minutes. Primary monocytes were incubated in the presence of propidium iodide (E) then intoxicated with LukAB (50ng/mL) and analyzed by flow cytometry. (F) Primary monocytes were intoxicated with LukAB (50ng/mL) and culture supernatants were analyzed for release of IL-18. Propidium iodide staining and IL-18 secretion are reported as a fraction of measurement in primary cells not treated with inhibitor. Bars represent the mean ± standard error of the mean for at least two independent experiments, each performed in triplicate. Asterisks indicate significance at a *p*-value of ≤ 0.05 by Tukey’s multiple comparisons post-test for 1-way or 2-way ANOVA, as appropriate.

The dose-dependent cytotoxic activity exhibited by the different *S*. *aureus* pore-forming toxins towards primary CD14+ human monocytes (Fig 2E) closely matched the results obtained by measuring FLICA-1 activation in these cells (Fig 4G). These data raised the question as to whether Caspase 1 was required for cell death, a feature of the pyroptotic inflammatory cell death pathway. To test this we transfected THP1 cells with siRNA targeting Caspase 1 or ASC, as a positive control. After 72 hours, Caspase 1 levels were noticeably reduced as determined by immunoblot analyses (Fig 6A). Knockdown of Caspase 1 had a slight reduction on LukAB-induced PI staining (Fig 6B) but nearly eliminated secretion of IL-1β and IL-18 (Fig 6C and 6D). Additionally, primary CD14+ human monocytes were also treated with two pharmacologic inhibitors of Caspase 1: VX-765, a potent and selective small molecule inhibitor of Caspase 1 [45], and zYVAD-FMK, a peptide-based inhibitor of Caspase 1 [46]. Following pretreatment with a dose titration of both inhibitors (up to 50 μM), primary monocytes intoxicated with LukAB showed no difference in cell death (Fig 6E), but both VX-765 and zYVAD-FMK suppressed IL-18 secretion (Fig 6F).

### LukAB promotes *S*. *aureus* escape from within human monocytes independent of NLRP3 or ASC, but dependent on CD11b

**Fig 7:**
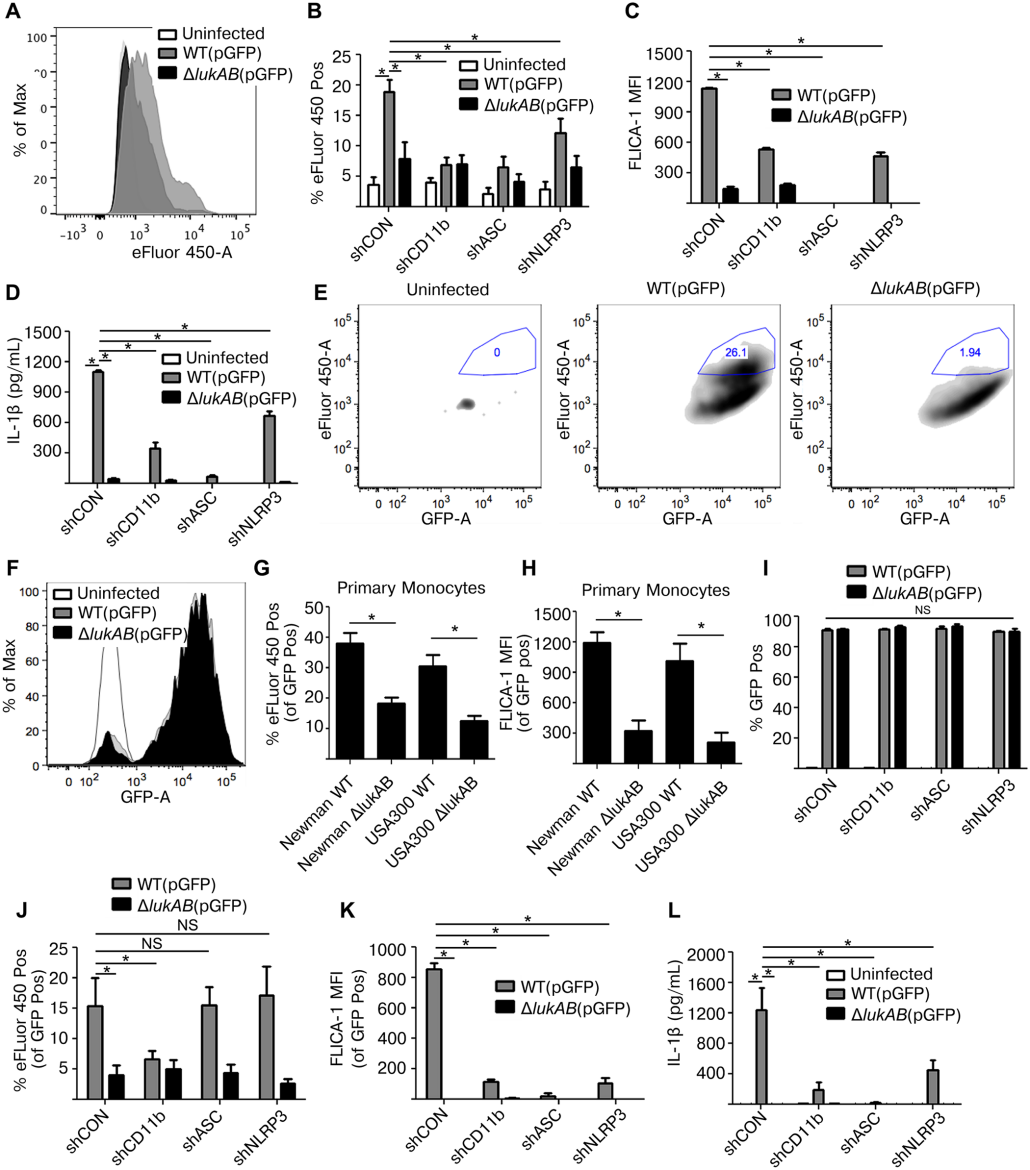
LukAB produced by extracellular or phagocytized *S*. *aureus* kills human monocytes. (A) THP1 cells were infected with *S*. *aureus* Newman strains at an MOI of 10 for 120 min under non-phagocytosing conditions (extracellular infection; see methods), stained with the fixable viability dye eFluor 450, a membrane damage and cell death marker, and analyzed by flow cytometry. Representative histogram is shown. (B-D) THP1 shRNA cells were infected with *S*. *aureus* Newman, MOI 10 for 120 min, followed by flow cytometry analysis for maximal eFluor 450 staining (B) and FLICA-1 activation (C). (D) Culture supernatants were collected from extracellular infections and analyzed for IL-1β secretion. (E and F) THP1 cells were infected with GFP-expressing *S*. *aureus* Newman strains at an MOI of 10 for 45 min under phagocytosing conditions (intracellular infection; see methods). (E) THP1 cells were stained with the fixable viability dye eFluor 450. After infection, THP1 cells were analyzed by flow cytometry and GFP-positive THP1 cells were selected, indicative of *S*. *aureus* phagocytosis. Maximal eFluor 450 incorporation was gated among GFP-positive cells, indicative of THP1 death by intracellular *S*. *aureus*. Very few cells appear in the first plot corresponding to the background autofluorescent uninfected cells. Representative plots are shown. (F) Representative histogram of GFP fluorescence in *S*. *aureus* infected and uninfected THP1 cells is shown. (G-H) Purified primary CD14+ human monocytes were infected with *S*. *aureus* Newman and USA300-BK18807 strains, along with respective isogenic *lukAB* mutants, at an MOI of 5 for 45 min then stained with eFluor 450 (G) or FLICA-1 (H), and analyzed by flow cytometry. Graphs reflect the fraction of cells that were GFP positive. (I-L) THP1 shRNA cells were infected under phagocytosing conditions with *S*. *aureus* Newman, MOI 10 for 45 min, and analyzed by flow cytometry for (I) GFP fluorescence indicating phagocytosis; (J) maximal eFluor 450 staining indicating cell death; and (K) FLICA-1 activation. (L) Culture supernatants were collected and analyzed for IL-1β secretion. Bars represent the mean ± standard error of the mean for at least two independent experiments, each performed in triplicate. Experiments with primary cell experiments include at least three independent donors. Asterisks indicate significance at a *p*-value of ≤ 0.05 by Tukey’s multiple comparisons post-test for 1-way or 2-way ANOVA, as appropriate.

We next sought to evaluate the contribution of ASC and NLRP3 in infection models with live *S*. *aureus*. THP1 cells were first infected with *S*. *aureus* Newman and the isogenic *lukAB*-deficient mutant, both constitutively expressing GFP, in the absence of opsonization (i.e. extracellular infection). Following infection, cells were stained with a fixable viability dye eFluor 450, a membrane damage and cell death marker, then analyzed by flow cytometry to determine the extent of THP1 cell death. In agreement with previous experiments using LDH and PI, THP1 cells infected with *S*. *aureus* lacking *lukAB* showed reduced eFluor 450 staining in comparison to THP1 cells infected with the wildtype strain (Fig 7A). In shRNA-transduced cell lines, we observed LukAB-dependent cell death (Fig 7B), FLICA-1 activation (Fig 7C), and IL-1β release (Fig 7D) in the control shRNA cell line. Each of these LukAB-mediated effects was also significantly reduced in the CD11b, ASC, and NLRP3 knockdown cell lines (Fig 7B–7D). The phenotypes observed in this extracellular infection model are consistent with our observations using purified toxins and culture filtrates.

LukAB targets CD11b to promote *S*. *aureus* escape from within human neutrophils [22,25]. We next sought to determine whether LukAB mediates similar escape from within human monocytes, and if NLRP3 or ASC contribute to this process. To evaluate cell death post-phagocytosis we modified our previously described method [25] to utilize GFP expressing strains and flow cytometric analyses. *S*. *aureus* Newman and the isogenic *lukAB*-deficient mutant were opsonized with human-serum and centrifuged in co-culture with THP1 cells to promote phagocytosis (Fig 7E–7L). Following phagocytosis, lysostaphin, a potent enzyme that kills *S*. *aureus* by degrading the cell wall [47], was added in combination with anti-LukA neutralizing antibodies to remove any remaining extracellular *S*. *aureus* and to block any residual extracellular LukAB [25]. We next quantified the proportion of cells that were both GFP positive and had maximal incorporation of eFluor 450, as an indication of THP1 cells terminally injured by intracellular *S*. *aureus*. We observed that post-phagocytosis, LukAB contributed to *S*. *aureus*-mediated membrane damage and cell death (Fig 7E). The distribution of GFP fluorescence in THP1 cells infected with WT *S*. *aureus* or the *lukAB*-deficient mutant were overlapping, indicating equal bacterial burden (Fig 7F). Importantly, *ex vivo* infections of primary CD14+ human monocytes with *S*. *aureus* Newman and a PVL+ USA 300 strain revealed that LukAB is indeed responsible for cell death (Fig 7G) and FLICA-1 activation (Fig 7H) post-phagocytosis in human monocytes.

Using this model, we next evaluated the contributions of CD11b, ASC and NLRP3 to *S*. *aureus*-mediated THP1 cell death. Post-infection, approximately 90% of THP1 cells were GFP-positive across all cell lines and between *S*. *aureus* strains (Fig 7I), indicating equivalent phagocytosis. We next quantified the proportion of THP1 cells killed by intracellular *S*. *aureus*. Remarkably, we observed that *S*. *aureus* induced THP1 cell death in a LukAB- and CD11b-dependent manner, but an ASC- and NLRP3-independent manner (Fig 7J). Furthermore, we observed that while death was ASC- and NLRP3-independent, LukAB-mediated FLICA-1 activation (Fig 7K) and IL-1β release (Fig 7L) occurred through CD11b, ASC, and NLRP3. Thus, taken together these results demonstrate that LukAB, when secreted by *S*. *aureus* in the extracellular milieu, activates the host NLRP3 inflammasome to promote killing. However, when secreted from within monocytes, LukAB activates the NLRP3 inflammasome and induces cell death independently of NLRP3 or ASC.

## Discussion

*Staphylococcus aureus* is a leading global cause of life threatening bacterial infections by virtue of its remarkable ability to invade practically all tissues in the human body, evade immune clearance, and subsequently proliferate. Its wide arsenal of virulence factors likely enables this broad tissue tropism and persistence. With regard to pore-forming toxins, many studies have focused on α-toxin (Hla) and Panton-Valentine leukocidin (PVL). Herein, we demonstrate that LukAB, the most recently identified *S*. *aureus* toxin [20,21], is a predominant cytolytic leukotoxin responsible for inducing programmed inflammatory cell death in human monocytes. Both purified LukAB and PVL have exquisite potency in activating the NLRP3 inflammasome and inducing inflammatory cell death in primary CD14+ human monocytes; a result consistent with high selectivity of these toxins toward human cells [3]. The dependency of *S*. *aureus* on any one toxin for survival and pathogenesis is likely tissue-, cell- and strain-specific. Unlike PVL, which is only encoded in about 15% of clinical isolates, LukAB is a part of the core genome of *S*. *aureus* and found in the vast majority of isolates (data publically available in NCBI sequenced *S*. *aureus* genomes).

Our data points to an important role for LukAB in mediating virulence through interacting with CD11b on human monocytes. In fact, during *ex vivo* infection of primary CD14+ human monocytes, LukAB seems to be the main factor produced by *S*. *aureus* responsible for targeting and killing monocytes. By activating the inflammasome in monocytes, LukAB is likely to induce uncontrolled production of pro-inflammatory cytokines such as IL-1β and IL-18, thus in theory worsening the outcome of *S*. *aureus* infections [23,48]. The presence of multiple toxins that activate a common host signaling pathway suggests there is selective advantage for *S*. *aureus* to activate the NLRP3 inflammasome, at least under some circumstances. Alternatively, when host activation of the NLRP3 inflammasome is detrimental to *S*. *aureus*, each of these toxins must also provide advantages to bacterial survival that are independent and outweigh the negative selective pressure inflammasome activation would exert on the bacteria.

Inflammasomes were originally discovered using THP1 cells [35], and much of the mechanistic details surrounding their activation and assembly have been performed in monocytes, monocytic cell lines, and macrophages. Only recently has the role of inflammasomes in other phagocytic and non-immune cells begun to be evaluated [49,50]. Neutrophils, which are critical phagocytes involved in controlling *S*. *aureus* infection [51], contain inflammasome components within secretory vesicles, tertiary granules, and also freely within the cytoplasm [52]. In contrast to monocytes and other cells, which process IL-1β primarily in a Caspase 1 dependent manner, neutrophils have been shown to have both Caspase 1—dependent and—independent mechanisms of IL-1β secretion. The Caspase 1-independent mechanisms involve serine proteases including elastase and proteinase 3 [52]. However, Caspase 1 has been shown to play a major role in IL-1β secretion by neutrophils in response to pore forming toxins including *Streptococcus pneumoniae* pneumolysin and nigericin and both toxins fail to activate Caspase 1 in mouse neutrophils lacking NLRP3 inflammasome components [53,54]. While evaluation of the role of NLRP3 and ASC in human neutrophils was beyond the scope of this work, the signals emanating from LukAB-CD11b interaction, both on the cell surface and in the phagosome, are likely conserved between monocytes and neutrophils. Thus it is likely that LukAB-dependent escape from neutrophils by *S*. *aureus* involves cell death pathways independent of ASC and NLRP3. Although mice lacking NLRP3 and other inflammasome components exist, they are not appropriate to assess the contribution of LukAB mediated escape from phagocytes, nor the role of inflammasome in this process due to the narrow host range for LukAB.

By using both genetic and pharmacologic approaches, we demonstrate that LukAB-mediated IL-1β and IL-18 secretion depends on Caspase 1. Consistent with a previous report describing the role of Caspase 1 activity in programmed necrotic cell death [55], we have found that genetic depletion of Caspase 1 by siRNA in THP1 cells and pharmacologic inhibition with zYVAD-FMK and VX-765 in primary CD14+ human monocytes does not alter LukAB-induced cell death. However, with these data we cannot rule out a role for Caspase 1 in programmed necrotic cell death. As previously reported [55], Caspase 1 can exist as a longer half-life executioner that persists through the time course of siRNA experiments or as a highly sensitive executioner resistant to total inhibition [56]. Our results do suggest that LukAB-mediated death shares common features with a pyroptotic mechanism but cannot be strictly classified as pyroptosis without further experimentation in human immune cells completely lacking Caspase 1.

In contrast to other *S*. *aureus* bi-component pore-forming toxins, which are found as water-soluble monomers in solution, LukAB is isolated as a dimer in solution [24,57]. The recently solved crystal structure of LukAB identified three salt bridges, unique to LukAB, that are involved in dimer formation [57]. While it is tempting to speculate that the “dimeric” nature of LukAB accelerates LukAB-mediated Caspase 1 activation and monocyte death, we observed similar potencies with PVL, which is not isolated as a dimer in solution nor targets CD11b [24]. Thus, these results suggest that the signals downstream of LukAB- and PVL-receptor binding could converge to potentiate Caspase 1 activation and monocyte death.

Another novel feature of LukAB is the activity of this toxin from within human neutrophil phagosomes, which facilitates bacterial escape and promotes cell killing [20,22,25]. We show here that this characteristic is also evident in primary CD14+ human monocytes. Using THP1 cells as a model, we determined that LukAB-mediated cell death from within monocyte phagosomes is CD11b-dependent, but NLRP3 or ASC-independent manner. In contrast, the extracellular model of infection revealed that *S*. *aureus* LukAB killed monocytic cells in a CD11b-, ASC- and NLRP3-dependent manner. Previously, LLO from *Listeria monocytogenes* has been reported to activate S10H3 dephosphorylation in addition to activating the NLRP3-ASC inflammasome [58], and escape of *L*. *monocytogenes* into the cytoplasm has been shown to cause infrequent bacterial lysis leading to AIM2-ASC inflammasome activation [59]. Our present study, however, represents the first of its kind showing an inflammasome-dependent cell death initiated by extracellular toxin, but an ASC-containing-inflammasome-independent cell death initiated specifically by intracellular toxin. A possible explanation for these observations is that additional signaling cascades that lead to cell death are engaged by LukAB-CD11b recognition within the phagosome as compared to LukAB-CD11b recognition on the cell membrane. On the contrary, a recent report suggested that phagocytosis of *S*. *aureus* by murine phagocytes triggers early activation of the NLRP3 inflammasome and Caspase 1; an event that is required for bacterial clearance [60]. The apparent discrepancy between our results can be attributed to the inability of LukAB to lyse murine cells [22,23].

The involvement of NLRP3 activation in human *S*. *aureus* infection is likely to be underestimated due to reliance on murine models which are resistant to LukAB-mediated lysis. LukAB binds to human CD11b with nearly 1000-fold higher affinity than to mouse CD11b [22]. Its clear however that in *ex vivo* models of infection of primary human phagocytes, LukAB is a dominant toxin involved in the targeting and killing of these important leukocytes [20–22,24,25]. Of note, a recent study revealed that children with invasive *S*. *aureus* disease exhibit a potent IgG response to LukAB [48], highlighting that LukAB is produced during human infection. These findings further support the notion that LukAB influences *S*. *aureus* pathophysiology in human infections. Ultimately, this study advances our understanding of how LukAB manipulates leukocytes, information critical to fully uncover how this virulence factor contributes to *S*. *aureus* infection.

## Materials and Methods

### Ethics statement

All protocols were conducted in accordance with National Institutes of Health guidelines for the care and use of human subjects. De-identified human blood packs were purchased from Gulf Coast Regional Blood Center or New York Blood Center. The use of the de-identified samples was reviewed by the UNC Office of Human Research Ethics, which determined that the proposed studies (Study #12–0024) do not constitute human subjects research as defined under federal regulations [45 CFR 46.102 (d or f) and 21 CFR 56.102(c)(e)(l)] and does not require further IRB approval. The New York City Blood Center obtained written informed consent from all participants involved in the study. This research was approved by the New York University School of Medicine institutional human subjects board.

### Mammalian cell lines

THP1 cells (ATCC TIB-202) were maintained in Roswell Park Memorial Institute medium 1640 (RPMI) medium (Cellgro) at 37°C with 5% CO_2_, where culture medium was supplemented with 10% heat-inactivated fetal bovine serum (FBS), penicillin (100 U/ml), and streptomycin (0.1 mg/ml). Transduced THP1 cells were maintained in 1.3 μg/ml puromycin. All experiments utilized cells that 2 days prior reached a density of approximately 0.8 x 10^6^ cells/ml before being split 1:2 with fresh media. Prior to infections or intoxications, the desired volume of cells was removed, centrifuged and suspended in fresh RPMI media and equilibrated for 1 hr at 37°C with 5% CO_2_. Unless specified, experiments were carried out in either 96-well or 48-well flat-bottom tissue culture treated plates. All experiments were conducted within approximately 1 month of thawing frozen cell stocks.

### Purification of primary CD14+ human monocytes

Human blood from leukopacks was diluted 1:2 with 1x phosphate buffered saline (PBS) supplemented with 0.1% bovine serum albumin (BSA) and 2mM EDTA or Hank's Balanced Salt Solution (HBSS). Diluted blood was layered over Ficoll-Paque (GE Healthcare Life Sciences) and centrifuged for isolation of buffy coats. Purified buffy coats were washed, counted, concentrated by centrifugation and labeled with CD14+ magnetic beads (Miltenyi Biotec). Cells were then washed to remove excess beads and separated per manufacturer’s instructions on a magnetic column. Purified primary CD14+ human cells were suspended in RPMI media with 10% (FBS) for 1 hour prior to intoxication experiments.

### Bacterial strains, culture conditions and generation of mutants

*S*. *aureus* isolate Newman [36] was used in all experiments as the “wild-type” (WT) strain (unless stated). *S*. *aureus* was grown on tryptic soy broth (TSB) solidified with 1.5% agar at 37°C. *S*. *aureus* cultures were grown in TSB or in RPMI (Invitrogen) supplemented with 1% Casamino Acids (RPMI+CAS), with shaking at 180 rpm. When appropriate, RPMI+CAS was supplemented with chloramphenicol (Cm) at a final concentration of 10 μg/ml.

Bacterial strains are listed in Table 1. Generation of the *S*. *aureus* Newman ΔΔΔΔ (Δ*lukAB*, Δ*lukED*, Δ*hlg*, Δ*hla*), and precursor strain *S*. *aureus* Newman Δ*lukAB*/Δ*lukED*/Δ*hlg* have been previously described [61]. To generate *S*. *aureus* Newman Δ*lukAB*/Δ*lukED*, a previous *lukAB* mutant [20] was transduced with a phage encoding *lukED*::*kan*. To generate green fluorescent protein (GFP) *S*. *aureus* strains Newman and USA 300-BK18807, and respective isogenic *lukAB* mutant, were transformed with *pOS1-P*_*sarA*_ *-sod RBS-*s*gfp*, a plasmid that constitutively and robustly produces superfolded GFP [62].

**Table 1:** *Staphylococcus aureus* strains used in this study.

Strain	Background	Description	Designation	Reference
VJT 1.07	Newman	WT/pOS1	Newman (*p*)	[20]
VJT 2.59	USA 200	WT	USA 200	[63]
VJT 2.84	USA 100	WT	USA 100	NARSA^a^
VJT 2.97	Newman	WT	WT or Newman	[36]
VJT 4.79	USA 400	MW2	USA 400	[64]
VJT 7.09	Newman	Δ*hlgACB*::*tet*	Δ*hlg*	[65]
VJT 8.16	Newman	Δ*lukED*	Δ*lukED*	[20]
VJT 8.91	Newman	Δ*lukAB*	Δ*lukAB*	[20]
VJT 9.72	Newman	Δ*lukAB*/pOS1	Newman Δ*lukAB* (*p*)	[20]
VJT 9.76	Newman	Δ*lukAB*/pOS1-*lukAB*	Newman Δ*lukAB* (*plukAB*)	[20]
VJT 10.21	USA 500-BK2395	WT	USA 500	[66]
VJT 12.61	USA 300-LAC	LAC	USA 300	[67]
VJT 15.15	USA 300-LAC	pOS1	LAC (*p*)	[20]
VJT 15.17	USA 300-LAC	Δ*lukAB*/pOS1	LAC Δ*lukAB* (*p*)	[20]
VJT 15.18	USA 300-LAC	Δ*lukAB*/pOS1-*lukAB*	LAC Δ*lukAB* (*plukAB*)	[20]
VJT 15.19	USA 400-MW2	pOS1	MW2 (*p*)	[20]
VJT 15.21	USA 400-MW2	Δ*lukAB/*pOS1	MW2 Δ*lukAB* (*p*)	[20]
VJT 15.22	USA 400-MW2	Δ*lukAB*/pOS1-*lukAB*	Mw2 Δ*lukAB* (*plukAB*)	[20]
VJT 21.05	USA 700	WT	USA 700	NARSA^a^
VJT 21.06	USA 800	WT	USA 800	NARSA^a^
VJT 21.08	USA 1100	WT	USA 1100	NARSA^a^
VJT 38.71	Newman	*pOS1sGFP-P_sarA_-sod RBS*	Newman GFP	This study
VJT 38.72	Newman	Δ*lukAB pOS1sGFP-P_sarA_-sod RBS*	Newman Δ*lukAB* GFP	This study
VJT 38.77	USA 300-BK18807	*pOS1sGFP-P_sarA_-sod RBS*	USA 300 GFP	This study
VJT 38.78	USA 300-BK18807	Δ*lukAB pOS1sGFP-P_sarA_-sod RBS*	USA 300 Δ*lukAB* GFP	This study

^a^NARSA, Network of Antimicrobial Resistance in *Staphylococcus aureus*.

### Purification of toxins from *S*. *aureus*

A construct to co-purify recombinant LukAB from *S*. *aureus* (*pOS-1-P*_*lukAB*_ *-sslukA-6His-lukA-lukB*) was generated as previously described [24] and transformed into Newman ΔΔΔΔ [61] to facilitate purification. This construct was also used to individually express toxin subunits from LukED, PVL, HlgABC [24]. Toxins were purified from *S*. *aureus* as previously described [24]. Briefly, strains were grown in TSB with 10 μg/ml chloramphenicol for 5 h at 37°C, 180 rpm, to an optical density at 600 nm (OD_600_) of approximately 1.5 (which represents 1 x 10^9^ CFU/ml). The bacteria were then pelleted, and the supernatant was collected and filtered. Nickel-nitrilotriacetic acid (NTA) resin (Qiagen) was incubated with culture supernatant, washed, and eluted with 500 mM imidazole. The protein was dialyzed in 1 × Tris-buffered saline (TBS) plus 10% glycerol at 4°C overnight and then stored at −80°C.

### Culture filtrate production

Culture filtrates were collected essentially as described previously [25]. Briefly, three-milliliter overnight cultures in RPMI+Cas were grown in 15-ml conical tubes held at a 45° angle and incubated at 37°C with shaking at 180 rpm. The following day, bacteria were subcultured at a 1:100 dilution and grown as described above for 5 h. Bacteria were then pelleted by centrifugation at 4,000 rpm [3220 x *g*] and 4°C for 10 min. Supernatants containing exoproteins were collected, filtered using a 0.2-μm filter, and stored at −80°C.

### Transmission electron microscopy

THP1 cells were intoxicated with culture filtrates (10% v/v) from WT *S*. *aureus* Newman, an isogenic *lukAB*-deficient mutant or culture media for 1 h at 37°C with 5% CO_2_. Cells were then fixed in 0.1 M sodium cacodylate buffer (pH 7.2), containing 2.5% glutaraldehyde and 2% paraformaldehyde for 2 h and post-fix stained with 1% osmium tetroxide for 1.5 h at room temperature, and en bloc stained with 1% uranyl acetate. The cells were dehydrated in ethanol then embedded in EMbed 812 (Electron Microscopy Sciences, Hatfield, PA). Semi-thin sections were cut at 1 μm and stained with 1% toluidine blue to evaluate the quality of preservation. Ultrathin sections (50 nm) were post stained with uranyl acetate and lead citrate and examined using Philips CM-12 electron microscope (FEI; Eindhoven, The Netherlands) and photographed with a Gatan (4 k × 2.7 k) digital camera (Gatan, Pleasanton, CA, USA).

### Cytotoxicity evaluated by measuring lactate dehydrogenase (LDH) release

For infection assays, *S*. *aureus* was cultured as described above for culture filtrate production then the bacterial pellet was washed twice with 5 ml of PBS. Bacteria were then normalized to an OD_600_ 1.0, which represents approximately 1.0 x 10^9^ CFU/ml using a Genesys 20 spectrophotometer (Thermo Scientific). Normalized *S*. *aureus* cultures were used to infect THP1 cells, seeded at 1 x 10^5^ cells/well, at a multiplicity of infection (MOI) of 50 in a final volume of 100 μl for 2 h at 37°C and 5% CO_2_. Controls for 100% viability were composed of THP1 cells without *S*. *aureus*, while controls for 100% THP1 lysis included the addition of Triton X-100 (0.2%). Following infection, cells were pelleted by centrifugation at 1,500 rpm [450 x *g*] at 4°C for 5 min and lactate dehydrogenase (LDH) release was assayed as a measure of THP1 viability using the CytoTox-ONE homogeneous membrane integrity assay (Promega) per manufacturer specifications. Briefly, 50 μl of culture supernatant was removed and added to wells containing 50 μl of LDH reagent and incubated for an additional 10 min at RT. Fluorescence was measured using a PerkinElmer Envision 2103 multilabel reader (excitation, 555 nm; emission, 590 nm), and data were normalized to 100% THP1 lysis.

THP1 cells were intoxicated with titrations of *S*. *aureus* culture filtrates (vol/vol) for 4 h at 37°C and 5% CO_2_. Controls for 100% viability were composed of cells with *S*. *aureus* growth medium (RPMI-CAS), while controls for 100% THP1 lysis included the addition of Triton X-100 (0.2%) in RPMI-CAS. THP1 viability was assayed by measuring LDH release as described above.

### Evaluation of cell death by propidium iodide staining

THP1 cells, seeded at 1 x 10^6^ cells/mL in 300 μL/well, were intoxicated with culture filtrates or LukAB in the presence of propidium iodide (2.5 μg/mL) for 60 minutes. Cells were fixed with a combination formaldehyde and methanol solution supplied by ImmunoChemistry Technologies. Fluorescence was measured by flow cytometry using an Accuri C6 flow cytometer (BD Biosciences).

### AlphaLISA for measuring cytokine expression

Culture supernatants from THP1 cells incubated with culture filtrates or LukAB were analyzed by alphaLISA for IL-1β, IL-18 and TNF-α according to the manufacturer’s protocol for short incubation (Perkin Elmer) with reduced volumes. Briefly, 1μL of each sample or standard was added to a separate well in a 384-well plate with 4uL of acceptor beads and cytokine antibody. After a 1-hour incubation at room temperature shielded from light, 5uL of streptavidin-conjugated donor beads was added to each well for 30 minutes. Luminescence was measured on an EnSpire Multimode Plate Reader (Perkin Elmer).

### Measuring Caspase 1 activation with FLICA

THP1 cells, seeded at 1 x 10^6^ cells/mL in 300uL/well, were intoxicated with culture filtrates or LukAB in the presence of the Caspase 1 inhibiting peptide FLICA-FMK bound to Alexa Fluor 660 (FLICA-1) (1:100 dilution) for 60 minutes. Cells were washed once with 1 x PBS and resuspended in 1 x PBS plus 8% fixative solution supplied by ImmunoChemistry Technologies. Fluorescence was measured by flow cytometry using an Accuri C6 flow cytometer (BD Biosciences).

### Immunoblot analysis

THP1 cells were washed with 1x PBS and lysed with RIPA buffer (50mM Tris, pH 7.4, 150mM NaCl, 0.1% SDS, 0.5% sodium deoxycholate, 1% NP-40, protease inhibitor cocktail) for a concentration of 1 x 10^7^ cell equivalents/mL. Lysate was spun at full speed in a mini centrifuge for 10 minutes at 4°C. Lysate supernatant was mixed with Laemmli sample buffer and heated to 95°C for 5 minutes. Samples were stored at -80°C until analyzed.

Samples were loaded at 1 x 10^5^ cell equivalents per well in a pre-cast 4–12% Bis-Tris SDS-PAGE gel (Bio-Rad Laboratories, Inc.). Electrophoresis was run at 120 volts for 100 minutes. Transfer was conducted using Trans-Blot Turbo Transfer System (Bio-Rad Laboratories, Inc.). Membranes were blocked with 5% milk solids or 5% BSA in 1 x TBS-T. Primary and HRP-conjugated secondary antibody incubation were performed overnight and for 1 hour, respectively, in blocking solution. Membranes were washed for 15 minutes three times after each antibody incubation. Membranes were developed using Pierce ECL Western Blotting Substrate or SuperSignal West Femto Chemiluminescent Substrate (Thermo Scientific) and imaged using a FluorChem E system (Protein Simple). All blots shown in the same figure are from the same experiment.

Antibodies used include anti-NLRP3, mAb (Cryo-2) at 1 to 1000 dilution (AdipoGen), anti-ASC, pAb antibody at 1 to 1000 dilution (Enzo Biosciences), anti-HMGB1 antibody (HAP46.5, ab12029) at 1:2000 dilution (Abcam), anti-Caspase 1 antibody (14F468) at a 1 to 1000 dilution (Novus Biologicals), anti-Actin antibody (SC-1615) at 1:5000 dilution (Santa Cruz Biotechnologies), Goat-anti Mouse antibody (SC-2005) at 1:5000 dilution (Santa Cruz Biotechnologies) and Goat-anti Rabbit antibody (SC-2004) at 1:5000 dilution (Santa Cruz Biotechnologies).

### Evaluation of CD11b levels on the surface of THP1

THP1 cells, seeded at 1 X 10^5^ cells/well, were stained with 1 ng/μl of APC-conjugated anti-CD11b (or isotype control) (Biolegend) in a final volume of 50 μl for 30 min on ice. Cells were washed once with 1x PBS + 2% FBS + 0.05% sodium azide (FACS buffer), suspended in 50 μl of FACS buffer, then analyzed using an LSR-II flow cytometer (Becton, Dickinson, BD).

### Infection assays evaluated by flow cytometry

THP1 infection assays under non-phagocytosing and phagocytosing conditions were modified from a previous study [25]. Briefly, GFP-expressing *S*. *aureus* Newman and the isogenic *lukAB*-deficient mutant were cultured and normalized to 1.0 x 10^9^ CFU/ml as described above. After normalization, bacteria were pelleted and suspended in phenol red-free RPMI with 10 mM HEPES Buffer (RPMI+HEPES) for non-phagocytosing conditions or opsonized with RPMI+HEPES supplemented with 20% normal human serum (NHS) for phagocytosing conditions. To promote opsonization, bacteria were incubated at 37^°^C with rotation for 30 min, centrifuged and pellet suspended in equivalent volume of RPMI+HEPES. Ninety-six well plates used for phagocytosing conditions were first coated with 20% NHS in RPMI+HEPES for 30 min at 37^°^C and subsequently washed with RPMI+HEPES.

Prior to infection, THP1 cells were primed with 500 ng/ml of purified *S*. *aureus* lipoteichoic acid (LTA) for 3 hrs, centrifuged at 1,500 rpm [450 x *g*] and 4°C then suspended in equivalent volume of RPMI+HEPES. THP1 cells, plated at 1 x 10^5^ cells/well, were infected with GFP *S*. *aureus* Newman or the isogenic *lukAB*-deficient mutant at an MOI of 10. For phagocytosing conditions, THP1 cells and bacteria were centrifuged at 1,500 rpm [450 x *g*] and 4°C for 7 min to promote synchronization of phagocytosis. Post-synchronization, cells were treated with 2.5 μg/ml of polyclonal anti-LukA antibody affinity purified from rabbit sera along and lysostaphin (40 μg/ml; Ambi Products LLC) to reduce effects of extracellular *S*. *aureus* and LukAB, then incubated at 37°C and 5% CO_2_ for 45 min. For non-phagocytosing conditions, bacteria were incubated at 37°C and 5% CO_2_ for 120 min.

Post-infection, cells were washed 2 times in 200 μl of PBS then stained with a 1:5,000 dilution of a Fixable Viability Dye (eFluor 450; Affymetrix eBioscience) and a 1:150 dilution of FLICA-1 in a final volume of 20 μl for 20 min on ice. Cells were washed 2 times in 200 μL of FACS buffer before being suspended in 40 μl of fixing buffer (1 x PBS + 2% paraformaldehyde + 2% FBS + 0.05% sodium azide) and analyzed using an LSR-II flow cytometer to measure GFP and eFluor 450 fluorescence.

## Supporting Information

S1 Fig ASC and NLRP3 contribute to *S*. *aureus* culture filtrate-mediated cytokine secretion and THP1 death. The indicated THP1 shRNA cells were intoxicated with culture filtrates (1% v/v) from *S*. *aureus* Newman. Culture supernatants were collected and analyzed for secretion of LDH release (A) and the indicated cytokines (B and C). Bars represent the mean ± standard error of the mean for at least two independent experiments, each performed in triplicate.
(TIF)

S2 Fig Media supplementation with KCL inhibits LukAB-mediated FLICA-1 activation and cell death. THP1 cells were incubated in media supplemented with an additional 25 mM NaCl, KCL or ChCl. Cells were incubated with FLICA-1 (A) or propidium iodide (B) then intoxicated with LukAB (50 ng/mL) for 1 hour and analyzed by flow cytometry. Bars represent the mean ± standard error of the mean for at least two independent experiments, each performed in triplicate.
(TIF)
