# Supplementary figures and images for: Staphylococcus aureus Leukocidin A/B (LukAB) Kills Human Monocytes via Host NLRP3 and ASC when Extracellular, but Not Intracellular
Source: PLoS Pathog. 2015 Jun 12;11(6):e1004970. doi: 10.1371/journal.ppat.1004970 (PMC4466499; doi:10.1371/journal.ppat.1004970)

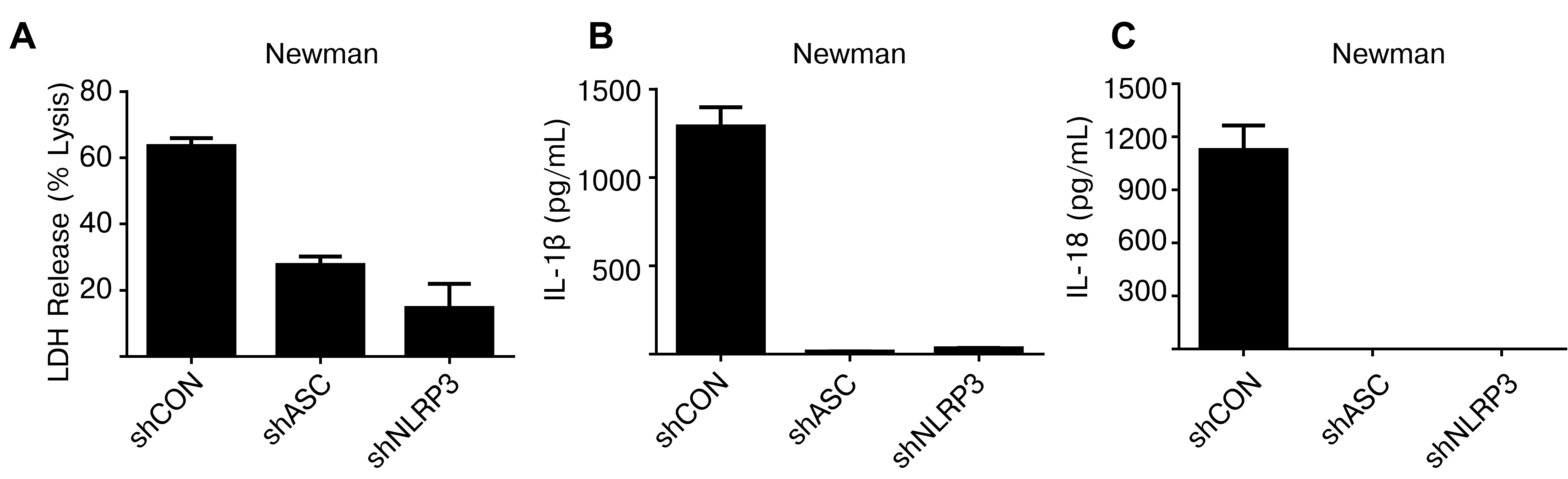

Supplement: S1 Fig — The indicated THP1 shRNA cells were intoxicated with culture filtrates (1% v/v) from S. aureus Newman. Culture supernatants were collected and analyzed for secretion of LDH release (A) and the indicated cytokines (B and C). Bars represent the mean ± standard error of the mean for at least two independent experiments, each performed in triplicate. (TIF) [file ppat.1004970.s001.tif]

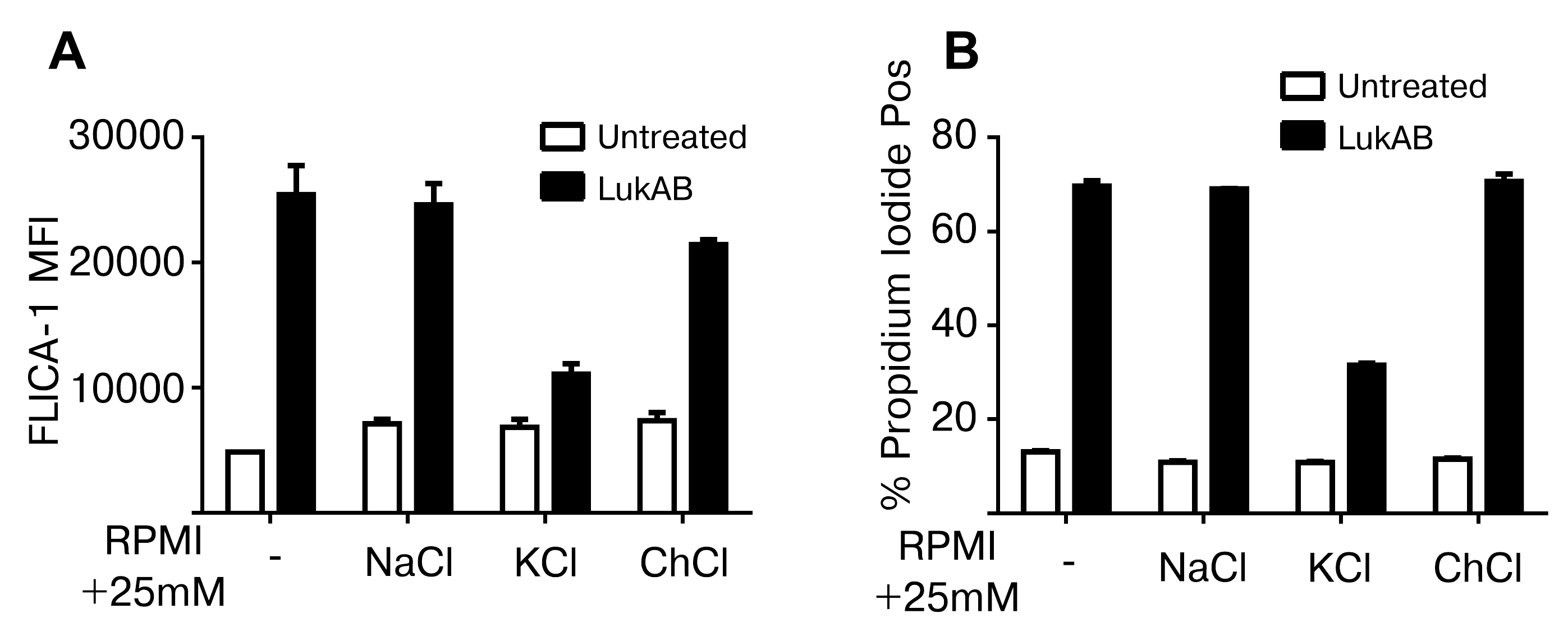

Supplement: S2 Fig — THP1 cells were incubated in media supplemented with an additional 25 mM NaCl, KCL or ChCl. Cells were incubated with FLICA-1 (A) or propidium iodide (B) then intoxicated with LukAB (50 ng/mL) for 1 hour and analyzed by flow cytometry. Bars represent the mean ± standard error of the mean for at least two independent experiments, each performed in triplicate. (TIF) [file ppat.1004970.s002.tif]
